# Collective contributions to the atomic Auger photoelectron coincidences on the (100), (110) and (111) facets of copper

**DOI:** 10.1038/s41598-025-06782-4

**Published:** 2025-07-21

**Authors:** Swarnshikha Sinha, Danilo Kühn, Fredrik. O. L. Johansson, Andreas Lindblad, Nils Mårtensson, Börje Johansson, Pavel A. Korzhavyi, Alexander Föhlisch

**Affiliations:** 1 Institut für Methoden und Instrumentierung der Forschung mit Synchrotronstrahlung, Helmholtz-Zentrum Berlin für Materialien und Energie GmbH, Albert-Einstein-Str. 15, 12489 Berlin, Germany; 2 Institut für Physik und Astronomie, Universität Potsdam, Karl-Liebknecht-Straße 24/25, 14476 Potsdam, Germany; 3 Uppsala-Berlin Joint Laboratory on Next Generation Photoelectron Spectroscopy, Albert-Einstein-Str. 15, 12489 Berlin, Germany; 4 Division of X-ray Photon Science, Department of Physics and Astronomy, Uppsala University, Box 516, 751 20 Uppsala, Sweden; 5 Physics Department and CSMB, Humboldt-Universität zu Berlin, Zum Großen Windkanal 2, 12489 Berlin, Germany; 6 Department of Physics and Astronomy, Uppsala University, P.O. Box 256, 751 20 Uppsala, Sweden; 7 Department of Materials Science and Engineering, KTH Royal Institute of Technology, Brinellvägen 23, 100 44 Stockholm, Sweden

**Keywords:** Synchrotron soft X-ray Spectroscopy, Auger Photoelectron Coincidences, Cu surface projected bandstructure, Screening of impurity states, Applied physics, Condensed-matter physics

## Abstract

For the Cu(100), Cu(110), and Cu(111) surfaces varying asymmetric line shapes are found for the atomic 3d^8^4s^2^ multiplet two-hole final state binding energies reached in MVV Auger photoelectron coincidence spectroscopy. Higher asymmetry for Cu(111) and Cu(110) in comparison to Cu(100) is caused by reduced dynamic screening for Cu(111) and Cu(110) in contrast to free electron like Cu(100). This is a consequence of the surface projected band gaps in Cu(111) and Cu(110) not present in Cu(100). We describe the distinct tailing in the experimental line shapes of the three Cu surfaces with first principles calculations of layer-dependent two-hole binding energy shifts, depth-dependent intensity distribution and Doniach-Sunjic asymmetry parametrization. These fundamental insights into the surface-specific electronic structure can advance the understanding of structure-reactivity relationships in Copper-based surfaces and catalysts.

## Introduction

The valence electronic structure of single crystal face-centered-cubic (fcc) Copper (Cu) is governed by the 3d and 4s electrons, leading to a rich band structure that has been studied to the highest accuracy both experimentally and computationally at different surface terminations^1–6^. Copper also serves for bench-marking experimental and computational electronic structure approaches, since its low electronic correlation in the ground state has led to an exceedingly good match between experimental valence electronic structure data and various computational electronic structure tools^7–13^. The electronic structure determination via the dispersing valence hole final state of angle resolved photoemission established a significant k-dependence of the spectral line shapes and the associated valence hole life-times^14–20^. Considerable attention has thus been devoted to the Cu(100), Cu(110) and Cu(111) faces and their remarkable differences in surface projected band structure^21,22^. The Cu(111) surface has a surface projected band gap around the ${\bar{\Gamma }}$ point^23–26^. The Cu(110) surface has at the Fermi level no band gap around the ${\bar{\Gamma }}$ point but surface projected band gaps at $\bar{X}$ and $\bar{Y}$^10,22^. In contrast, Cu(100) has no band gap. The significantly different surface projected densities of states cause a strong variation of the surface resonance states^27–29^.

The valence electronic structure of fcc Cu can also be approached in the framework of atomic configuration interaction (CI). Here, the [Ar]3d^10^4s^1^ electron configuration of a closed 3d-shell and a singly occupied 4s shell is dominant. In addition, the [Ar]3d^9^4s^2^ electron configuration contributes. Experimentally, Auger photoelectron coincidence spectroscopy (APECS)^30–34^ is highly sensitive to these CI contributions which govern the localization and sharing of d-electrons in mixed valence functional materials. For fcc Cu, 2p $\textrm{L}_3$VV APECS has shown that the metallic screening process leads to a 3d^10^4s^2^ valence occupation in the presence of the $\textrm{2p}^5$ core vacancy and the L_3_VV decay of this metallic screened 2p^5^3d^10^4s^1^ core-hole state further leads to a 3d^8^4s^2^ atomic multiplet final state^35^.

In this work we investigate how the different surface projected band structures of the Cu(100), Cu(110) and Cu(111) surfaces influence the degree of atomic localization and how they modify the dynamic screening response of the 3d^8^4s^2^ two-hole final state, which is reached in the Auger decay of the Cu 3p core hole detected with Auger photoelectron coincidence spectroscopy. We find dominant atomic Cu 3d^8^4s^2^ multiplet features for all faces of fcc Cu. Layer dependencies of the two-hole Cu 3d^8^4s^2^ binding energies are obtained within Z+2 impurity model^36,37^ from first-principles slab calculations for the Cu(100), Cu(110), and Cu(111) surfaces. We quantify an enhanced asymmetry of the 3d^8^4s^2^ Auger peaks from Cu(100) to Cu(110) and Cu(111) and interpret this trend as the decreasing metallic screening probability from the Cu(100) to Cu(110) and Cu(111) surfaces. This intricate understanding of electronic structure provides mechanistic insights into facet-dependent catalytic behavior, across Cu surfaces influencing reaction mechanisms and surface chemistry.

## Methods

The APECS measurements were conducted at the CoESCA (Coincidence Electron Spectroscopy for Chemical Analysis) endstation^34^ at the UE52-PGM beamline at BESSY II of the Helmholtz-Zentrum Berlin. Three Cu single crystals, Cu(100), Cu(110) and Cu(111), (99.99 %) from MaTecK GmbH were used. The crystals were cleaned by repeated cycles of Ar^+^ sputtering and annealing at 900K, until a sharp LEED pattern was obtained and no elements other than Copper were detected within the core level photoelectron survey spectrum. The experiments were performed under UHV conditions ($2\times 10^{-10}$ mbar). The photon energy h$\nu$ was set to 380±0.1 eV with near horizontal polarisation at an incidence angle of 5° between surface normal and photon beam. We are thus 310 eV above the Cu M-edge (3p) ionization potential in the sudden limit of ionization. In addition, the ArTOF energy window is proportional to kinetic energy and inverse proportional to the resolving power. 380 eV is thus an optimum energy with a window of 12 eV and experimental energy resolution of 0.3 eV. The MVV Auger photoelectron coincidence detection allows also significantly higher surface sensitivity and spectral resolution over the Cu LVV detection (see supplementary information)^38^. Fcc Cu has a lattice constant of 0.359 nm^39,40^ (interlayer spacing of 0.21 nm for the Cu(111) surface). Cu MVV Auger Electrons at 61 eV kinetic energy yield an inelastic mean free path of 0.497 nm making them highly surface sensitive in contrast to the Cu $\textrm{L}_3$VV Auger electrons at 912 eV kinetic energy with an inelastic mean free path of 1.56 nm (see supplementary information for full treatment of the Cu(111) surface and layer dependent signal for Cu MVV and Cu LVV)^38,41^.

The two Angle resolved Time of Flight (ArTOF) electron analyzers of the CoESCA endstation were set up in the following way to acquire coincidence spectra: ArTOF 1 detected the Cu MVV Auger decay electrons with an angular acceptance of 30° and ArTOF 2 the Cu 3p photoelectrons with an angular acceptance of 56°. Within the horizontal polarization plane, the central axis of ArTOF 2 is 59° off the sample normal and the central axis of ArTOF 1 is 49° off the sample normal on the opposite side. Due to the high detection efficiency of the two ArTOF electron analyzers in combination with the Pulse Picking by Resonant Excitation method^42^ at BESSY II, a high true-to-accidental ratio is achieved in the Cu MVV APECS measurements. For data acquisition above the single event limit the “true” coincidence events are accompanied by a fraction of “accidental” coincidences: The true coincidences are pairs of photoelectrons and Auger electrons originating from a single atomic ionization event. The accidental coincidences are electrons which arrive at the detectors within the acquisition window, but are from different ionization events. In our data acquisition scheme, a time stamp for each electron event on one of the two detectors is stored. Hence, one can extract a coincidence data set with electron pairs arising from the same photon pulse (same time stamp) which comprises “true” and “accidental” events. Additionally, a second dataset is extracted from the same measurement consisting of electron pairs where one electron is detected in one pulse and the other electron is detected in either the preceding or following pulse. Since electrons from different pulses can not originate from the same ionization event, this second dataset contains exclusively accidental coincidences. The rate of these cross-pulse accidental coincidences is the same as the rate of accidental coincidences occurring within a single pulse. This allows to accurately remove the contribution from accidental coincidences in the primary dataset by subtracting the two-dimensional coincidence map of the second dataset (only accidental coincidences) from the coincidence map of the primary dataset (true and accidental coincidences)^34^.

## Results and discussion

**Fig. 1:**
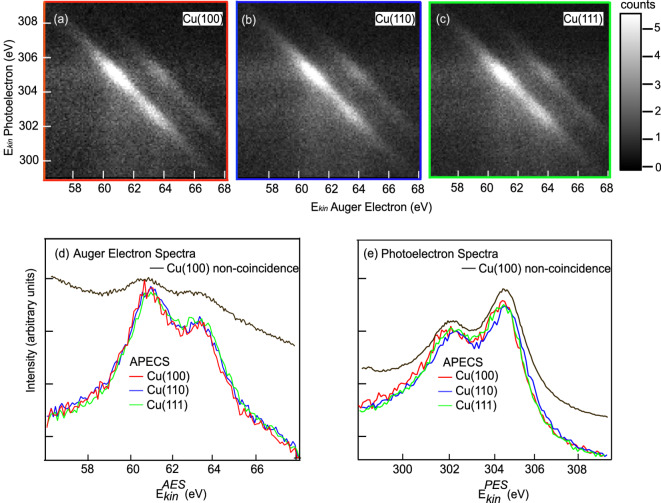
Auger photoelectron Copper 3p-MVV Coincidence maps for the Cu(100) (**a**), Cu(110) (**b**), and Cu(111) (**c**) surfaces. True coincidences are obtained by subtraction of accidental coincidence counts described in the main text. The linear greyscale gives the true coincidence count per pixel normalized to the average pixel count. Panel (**d**) shows M_23_VV Auger spectra obtained by integration along the entire photoelectron energy scale of the 2D coincidence map (integration region indicated with colored horizontal lines at the edges in (**a**), (**b**), (**c**)). Panel (**e**) shows photoemission spectra of the 3p_3/2_ and 3p_1/2_ doublet obtained by integration of the Auger energy scale along the entire 2D coincidence map (integration region indicated with colored vertical lines at the edges in (**a**), (**b**) and (**c**)). These Auger and Cu 3p photo electron spectra do not show any clear distinction between the three surfaces of Cu. Panels (**d**) and (**e**) also give non-coincidence Auger electron and photoelectron spectra, that include an additional multiple-scattering background.

Figure 1 a–c, shows maps of true coincidences of Auger/Photoelectron pairs plotted against their kinetic energies ($E^{AES}_{kin}, E^{PES}_{kin}$), referenced to the Fermi level (see supplementary information, which includes^43^). The experimental settings did not vary during the acquisition of data for the maps. The greyscale is linear and gives the true coincidence count per pixel normalized to the average pixel count. We observe diagonal features within the 2D coincidence maps of all three surfaces in Fig. 1a–c. The main line shows the ^1^G final state of the atomic $\textrm{3d}^8$ multiplet. The faint diagonal feature parallel to the main and shifted by about 3 eV to higher Auger kinetic energy signifies the ^3^F final state of the atomic $\textrm{3d}^8$ multiplet. These diagonal features reflect the localized atomic nature of the Cu 3p-MVV process where emission of the photoelectron and the Auger electron remains a coherent process preserving the energy sum^44^. Fig. 1d and e show Auger electron spectra and photoelectron spectra which are obtained by integrating the 2D maps from a–c over the photoelectron energies (d) or the Auger electron energies (e), respectively. The photoelectron spectra show the Cu $\textrm{3p}^{5}_{3/2}$ and Cu $\textrm{3p}^{5}_{1/2}$ spin-orbit components. As a direct consequence of the total energy conservation in the photo excitation/Auger decay process $\left( {E_{{kin}}^{{AES}} + E_{{kin}}^{{PES}} = h\nu - E_{{2H}} } \right)$,where $E_{2H}$ is the two-hole final state binding energy, the atomic-like two-hole final state peaks are in the Auger spectrum broadened by the core-hole lifetime $\left( {\Gamma _{{PE}} } \right),$ resulting in a Auger line width $\left( {\Gamma _{{AE}} } \right)$ being the sum of the natural lifetime width of the two-hole state $\left( {\Gamma _{{2H}} } \right)$ and the core hole lifetime width $\left( {\Gamma _{{AE}} = \Gamma _{{2H}} + \Gamma _{{PE}} } \right)$^45^. Because of this, the Auger spectra in Fig. 1d are mainly resembling the shape of the photoemission spectra of Fig. 1e in opposite energy direction, rather than the multiplet of the two-hole final states. Hence, the two-hole final states can not be accurately measured with conventional Auger Spectroscopy, in particular when the decay process is rapid (large core-hole life time broadening) as is the case in the Cu3p–MVV Super Coster-Kronig process. Additionally, non-coincidence photoelectron and Auger electron measurements suffer from secondary inelastic backgrounds, also shown for comparison in panels d) and e) of Fig. 1. These mechanisms have been previously discussed generally by^45,46^.

**Fig. 2:**
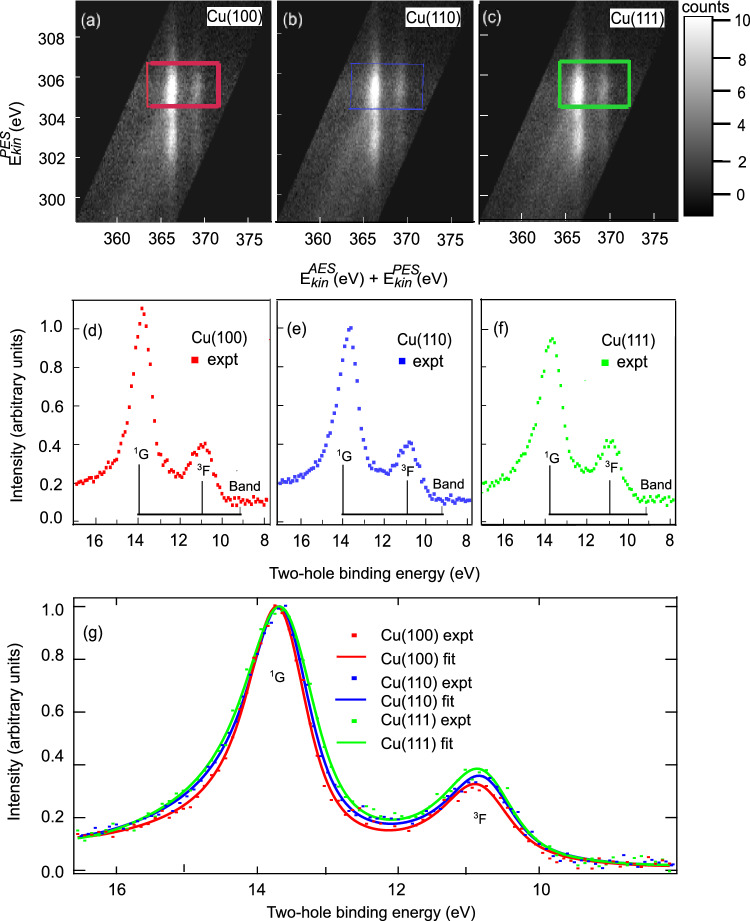
Transformation of the Auger photoelectron Cu 3p–MVV coincidence data from Fig. 1 onto the two-electron kinetic sum energy $E^{AES}_{kin}$(eV)+ $E^{PES}_{kin}$(eV) and the photoelectron kinetic energy $E^{PES}_{kin}$(eV). Panels (**a**), (**b**) and (**c**): Cu(100), Cu(110), Cu(111), respectively. Linear greyscale of true coincidence count per pixel normalized to the average pixel count. Panels (**d**), (**e**), and (**f**): Projected spectral distributions on the two-hole binding energy scale relative to the Fermi level (integration of Cu $\textrm{3p}_{3/2}$ region within each box: red Cu(100), blue Cu(110), and green Cu(111)). Panel (**g**): Direct comparison of the ^1^G and ^3^F atomic multiplet line shapes of the Cu 3d^8^4s^2^ final state for the three surfaces: Removal of band contribution and normalization to ^1^G peak height.

The coincidence data sets from Fig. 1a–c are thus converted in Fig. 2 a–c, to maps showing the sum of the kinetic energies of the Auger and photoelectron pairs $E^{AES}_{kin} + E^{PES}_{kin}$ and the kinetic energy of the photoelectron $E^{PES}_{kin}.$ These maps show the localized atomic signatures at constant sum energy $E^{AES}_{kin} + E^{PES}_{kin}.$ In addition, faint diagonal features stem from true coincidence events in which the photoelectron has undergone inelastic scattering.

The energy loss tails of the photoelectrons and Auger electrons appear in Fig.1 at constant Auger and photoelectron energies, respectively. We now obtain two-hole spectra by integrating the intensity over the photoelectron kinetic energy $E^{PES}_{kin}$ within the integration box. We chose the integration region with focus on $\textrm{3p}_{3/2}$ region so that we remain unaffected by the inelastic scattering tails mixing in from the Cu $\textrm{3p}_{1/2}$ region when we are in the two-electron kinetic sum energy $E^{AES}_{kin}$ (eV)+ $E^{PES}_{kin}$ (eV) scale. In addition, we convert the two-electron sum energy scale $E^{AES}_{kin} + E^{PES}_{kin}$ into the two-hole binding energy scale $E_{2H}$ = h$\nu$ -$E^{PES}_{kin}$-$E^{AES}_{kin}$ with $h\nu =380\pm 0.1$ eV (see supplementary information). The resulting two-hole spectra are shown in Fig. 2 d–f, for the three surfaces. Most prominent are the ^1^G and ^3^F atomic multiplets of the Cu 3d^8^4s^2^ final state reached in M_3_VV Auger decay following the initial Cu $\textrm{3p}_{3/2}$ photoionization. The atomic multiplet has been previously reported^47^ with its angular anisotropies^48^.

We note, that the Cu 3d^8^4s^2^ final state reached via the M_3_VV has different band-like contributions Fig.2 d–f as seen in the ratios of ($\it Intensity (^1G)$/*Intensity*(*Band*)) for Cu(111) as 7.06, Cu(110) as 7.82, and Cu(100) as 9.62. This reflects the closed packed nature of the Cu(111) surface with higher overlap of itinerant states in comparison to the more open packed Cu(100) surface with lesser overlap and Cu(110) being more similar in packing to Cu(111).

In order to analyze the Cu 3d^8^4s^2^ ^1^G and ^3^F atomic multiplets for the three surfaces, we remove the band contribution by an offset and normalize to the ^1^G peak height. The ^1^G peak heights are determined by unconstrained fitting (refer to supplementary information). Fig.2, panel (g) shows within experimental accuracy identical two-hole binding energies accompanied by an intensified broadening and asymmetric tailing of the Cu(110) and Cu(111) surfaces compared to the Cu(100) surface. To quantify the distinct line shapes of the Cu 3d^8^4s^2^ ^1^G and ^3^F atomic multiplets for the three surfaces, we first calculate the layer-dependent two-hole final state impurity energies for the respective Cu(100), Cu(110), and Cu(111) surfaces from first principles, tabulated in (Table 1).

The Vienna Ab-initio Simulation Package (VASP)^49^ was used, based on density functional theory (DFT) in the generalized gradient approximation (PBE96 exchange-correlation functional^50^). Supercell models of the three low-index surfaces of clean Cu were constructed assuming a slab geometry and periodic boundary conditions. The slabs possessing central symmetry contain 9 atomic layers for Cu(100), 11 atomic layers for Cu(110), and 10 atomic layers for Cu(111) separated by a vacuum region of about 20 Å; the surface unit cell for each atomic layer was doubled in the two in-plane dimensions to contain 4 atoms (slab model with $N/L=4$) or doubled twice (slab model with $N/L=16$). The atomic positions in the three (four for Cu(111) slab) inner layers representing the bulk, as well as the in-plane lattice parameters, were kept fixed at the ideal positions of bulk fcc Cu with a lattice parameter of 3.63 Å^40,51,52^. The atomic positions of all the other layers in the three slabs modeling clean Cu surfaces were optimized (relaxed). After the relaxation, surface core-level binding-energy shifts were computed as segregation energies of equivalent (Z+2) Ga impurities^36,37^ in the three outermost atomic layers (two such impurities were placed symmetrically at both surfaces of each slab), relative to the configurations where the Ga impurities were placed (at the maximum possible distance from one another) into the inner layers of the corresponding slabs (see supplementary for more information). In the total energy calculations, we employed projector augmented wave (PAW) type pseudopotentials^53,54^ Cu_GW and Ga_d_GW, designed to be more accurate for excited states calculations than the standard PAW potentials, together with a plane-wave basis set with an energy cutoff of 500 eV. For Brillouin zone integration, a 13 × 13 × 5 Monkhorst-Pack mesh of special k-points^55^ was used, with a Gaussian smearing of 0.1 eV.

**Table 1:** Layer dependence Surface Core Level Shifts (SCLS) of Cu, obtained with two different slab models, first model with surface unit cells containing $N/L=4$ atoms per atomic layer for the three Cu surfaces and second model with $N/L=16$, using VASP, DFT calculations. See Fig.3 for $N/L=4$ slab geometries and layer definition. The two-hole final state is described in the equivalent (Z+2) impurity approximation – effectively as a Ga impurity in Cu. For Cu(100), Cu(110), and Cu(111) surfaces, the energy shifts $\Delta E$ of layers 1, 2, and 3 are given relative to the bulk energy set to 0 eV for both the slab models.

Surface	*N*/*L*	$\Delta E$ (eV)		
		Layer 1	Layer 2	Layer 3
Cu(100)	4	− 0.347	+ 0.067	+ 0.003
	16	− 0.368	+ 0.052	+ 0.002
Cu(110)	4	− 0.448	− 0.109	+ 0.010
	16	− 0.404	− 0.103	+ 0.024
Cu(111)	4	− 0.419	+ 0.039	− 0.016
	16	− 0.460	+ 0.012	− 0.023

**Fig. 3:**
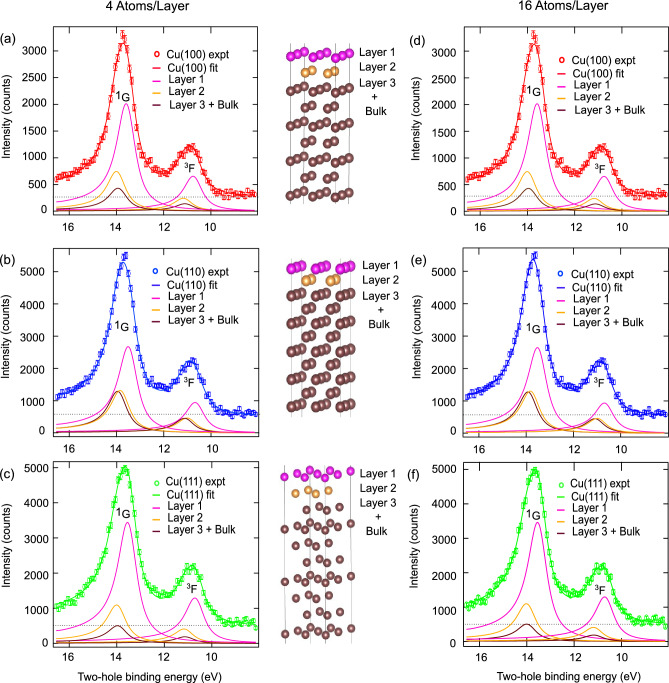
Panels (**a**), (**b**), (**c**), (**d**), (**e**) and (**f**) show spectra of Cu 3d^8^4s^2^ two-hole final states of Cu(100), Cu(110) and Cu(111) with the fits obtained using Doniach-Sunjic line shapes, layer-dependent energy shifts and depth-dependent intensity contributions of Layer 1, 2 and Layer 3 merged into bulk for two different slab models as described in the main text. Fitting results given in Table 2. Structure and layer definition from cutouts of geometry optimized slab calculations are shown.

From the layer-dependent Surface Core Level Shift (SCLS) energies of the two slab models in Table 1 we note that the surface Layer 1 and the subsurface Layer 2 are distinctly different from the bulk energy, which is set to 0 eV. Whereas Layer 3 has already converged to bulk like values and is from here on included into the bulk for the slab models: first with $N/L=4$ atoms per atomic layer in their unit cell and second with $N/L=16$ atoms per atomic layer in their unit cell . We can now model the experimental spectral shape of the atomic multiplet structure combining the layer-dependent SCLS energy shifts, an depth-dependent intensity model based on the universal curve of electron mean free path^38^ and an asymmetric line shape to the lower kinetic energy^56–59^.

This asymmetry to low kinetic energy is caused by the multibody screening response of the metallic system to the vacancy final state impurity in the Cu MVV process: low energy electron-hole shake-up continua screen the vacancy states in addition to discrete shake-up excitations^56^. The strength of shake-up processes of impurity sites coupled to metallic continua is strong for weak coupling and reduced for strong coupling^60,61^. Mahan, Noziere and DeDominicis have established theoretically the trends of main line line-shape asymmetry due to dynamic screening as a function of metallicity for deep and shallow hole states^58,59^. Doniach and Sunjic established a widely used analytical line-shape parametrization^57^. The Doniach-Sunjic asymmetry parameter $\alpha _{DS}$ relates to the Mahan dynamic screening approach and the Thomas-Fermi approximation as $\alpha _{DS} = 0.083 r_s c_q^2$ ($r_s$ is the Wigner Seitz radius in Bohr $a_o$ and $c_q$ the effective charge of the hole)^56,57^. This sets the lower limit of asymmetry reached in a hypothetical free metal. For “real” simple metals the following asymmetries are found: Be($\alpha _{DS}$=0.05), Na($\alpha _{DS}$=0.19), Mg($\alpha _{DS}$=0.12), Al($\alpha _{DS}$=0.11)^56^. Asymmetry to high kinetic energy can be caused by post-collision-interaction (PCI)^62–64^ for threshold excitation or as shake-down features in Van-der-Waals coupled systems^56^. Since we are in the sudden limit of ionization (310 eV above the threshold) and in metallic Cu, both effect do not play a role. We now determine the Doniach-Sunjic line-shape asymmetry parameter $\alpha _{DS}$ to characterize the collective multi-body screening response of MVV Cu impurity sites screened on the Cu(111), Cu(110) and Cu(100) surfaces with their different surface projected band structures. The depth-dependent intensity contributions for Cu(100), Cu(110) and Cu(111) use the interlayer spacing of Cu (see supplementary information, which includes references^41,65^) and the surface sheet density (no. of atoms/Area), derived from the VASP slab model calculations.

**Table 2:** Results of the Cu 3d^8^4s^2^  ^1^G and ^3^F atomic multiplet fit with Doniach-Sunjic line shape for slab models with $N/L=4$ and $N/L=16$ atoms per atomic layer in their unit cell (Gaussian FWHM at experimental bandwidth set to 0.3 eV, lifetime broadening equal for all Cu surfaces with a Lorentzian FWHM of 0.76 ± 0.01 eV), for both slab models. For Layer dependent SCLS energy shifts see Table 1 and depth-dependent intensity contribution see the supplementary information. Relative energy shifts ±0.01 eV. Experimental energy uncertainty 100 meV.

Surface	*N*/*L*	Asymmetry	Layer 1	Layer 1	$\frac{Intensity (^3F)}{Intensity (^1G)}$
		Parameter $\alpha _{DS}$	^1^G(eV)	^3^F (eV)	
Cu(100)	4	0.123	13.54	10.71	0.32
	16	0.121	13.54	10.71	0.32
Cu(110)	4	0.148	13.45	10.62	0.35
	16	0.153	13.46	10.63	0.35
Cu(111)	4	0.153	13.48	10.65	0.37
	16	0.150	13.48	10.65	0.37

The spectral fit results are tabulated in Table 2 for slab models with $N/L=4$ and $N/L=16$ atoms per atomic layer in their unit cell shown in Fig. 3 (a)–(f). (Fitting is done with Igor Pro Version 8(Windows). The depth-dependent intensity contributions of each Layer are given as input parameters see the supplementary information and^38^. Experimental and lifetime broadening are given by Gaussian and Lorentzian contributions. Gaussian Full Width at Half Maximum (FWHM) is the experimental bandwidth, 0.3 eV for all Cu surfaces for the two slab models. The lifetime broadening is a global variable for the ^1^G and ^3^F peaks for all three surfaces and found to be 0.76 ± 0.01 eV (FWHM) for both of the slab models. The energy splitting of the ^1^G and ^3^F components is linked between the three Cu surfaces and has a value of 2.83 ± 0.01 eV for both of the models. The Cu 3d^8^4s^2^ ^1^G and ^3^F peak positions, the Doniach-Sunjic asymmetry parameter $\alpha _{DS}$ and the intensity ratios of the ^3^F and ^1^G components are summarized in Table 2 for the three Cu surfaces.

The two-hole binding energy of the Cu 3d^8^4s^2^ multiplet is unvaried for the Cu surfaces, but a significant variation in asymmetry is extracted from our fit approach as the Doniach-Sunjic asymmetry parameter for $N/L=4$ slab model is found to be $\alpha _{DS}=0.123 \pm 0.01$ for Cu(100), $\alpha _{DS}=0.148 \pm 0.01$ for Cu(110), and $\alpha _{DS}=0.153 \pm 0.01$ for Cu(111). For $N/L=16$ slab model, the Doniach-Sunjic asymmetry parameters are found to be $\alpha _{DS}=0.121 \pm 0.01$ for Cu(100), $\alpha _{DS}=0.153 \pm 0.01$ for Cu(110), and $\alpha _{DS}=0.149 \pm 0.01$ for Cu(111). In comparison to the previously discussed free-electron simple metal systems Be($\alpha _{DS}$ =0.05), Na($\alpha _{DS}$ =0.19), Mg($\alpha _{DS}$ =0.12), Al($\alpha _{DS}$ = 0.11)^56^), the low $\alpha _{DS}=0.12 \pm 0.01$ of Cu(100) indicates an almost free-electron like mobility, that is reduced for Cu(110) and Cu(111) with $\alpha _{DS}=0.15 \pm 0.01$ due to the significant surface projected band gaps of the Cu(111) and Cu(110) surfaces in contrast to Cu(100). Cu(111) has a surface projected band gap at the ${\bar{\Gamma }}$ point, and Cu(110) at $\bar{X}$ and $\bar{Y}$), Cu(100) is without a surface projected band gap^10^. This variation in the collective, dynamic screening response of the Cu 3d^8^4s^2^ impurity state on the three Cu fcc surfaces causes the variation of asymmetry. Both slab models converge to similar values for asymmetry and two-hole binding energies, suggesting that the number of atoms per atomic layer in the unit cell, within the (Z+2) approximation, does not significantly affect the asymmetry. This indicates that the model to extract asymmetry is sturdy and retains its key characteristics despite variations in the atomic layer configuration.

## Conclusion

In conclusion, we observe for Cu(100), Cu(110), and Cu(111) dominant 3d^8^4s^2^ multiplet final states at identical two-hole binding energies in MVV Auger photoelectron coincidence spectroscopy. In addition, higher asymmetric line shapes are found for Cu(111) and Cu(110) in comparison to the Cu(100) surface. This trend reflects the varied surface projected band structures: Highest dynamic screening probability on the free electron like Cu(100) surface and lesser screening on the surface projected band-gapped Cu(110) and Cu(111) surfaces. A full spectral model is given based on first principles calculations of layer-dependent two-hole binding energy shifts, the depth-dependent intensity distribution and the Doniach-Sunjic asymmetry parametrization, providing a fundamental framework for understanding surface-specific electronic interactions. These findings have important implications for facet-dependent surface chemistry and their behavior in heterogeneous catalysis.

## Supplementary Information

Supplementary Information.

## Data Availability

The datasets used and analysed during the current study are available from the corresponding author on reasonable request.
