# Supplementary material for: Collective contributions to the atomic Auger photoelectron coincidences on the (100), (110) and (111) facets of copper
Source: Sci Rep. 2025 Jul 21;15:26411. doi: 10.1038/s41598-025-06782-4 (PMC12280130; doi:10.1038/s41598-025-06782-4)
Supplement: Supplementary file 1 — Supplementary Information. [file 41598_2025_6782_MOESM1_ESM.pdf]

Collective contributions to the atomic Auger photoelectron coincidences on the (100), (110) and (111) facets of Copper

Swarnshikha Sinha[1,2,3*], Danilo Kühn[1,3], Fredrik. O. L. Johansson[3,4], Andreas Lindblad[3,4], Nils Mårtensson[3,4], Börje Johansson[5,6], Pavel A. Korzhavyi[7], and Alexander Föhlisch[1,2,3*]

[1]Institut für Methoden und Instrumentierung der Forschung mit Synchrotronstrahlung, Helmholtz-Zentrum Berlin für Materialien und Energie GmbH,Albert-Einstein-Str. 15, 12489 Berlin, Germany
[2]Institut für Physik und Astronomie, Universität Potsdam, Karl-Liebknecht-Strasse 24/25, 14476 Potsdam, Germany
[3]Uppsala-Berlin Joint Laboratory on Next Generation Photoelectron Spectroscopy, Albert-Einstein-Str. 15, 12489, Berlin, Germany
[4]Division of X-ray Photon Science, Department of Physics and Astronomy, Uppsala University, Box 516,SE-751 20, Uppsala, Sweden
[5]Humboldt-Universität zu Berlin, Physics Department and CSMB, Zum Großen Windkanal 2, D-12489 Berlin, Germany
[6]Department of Physics and Astronomy, Uppsala University, P.O. Box 256, 751 20 Uppsala, Sweden
[7]Department of Materials Science and Engineering, KTH Royal Institute of Technology, Brinellvägen 23, 100 44 Stockholm, Sweden
[*]swarnshikha.sinha@helmholtz-berlin.de
[*]alexander.foehlisch@helmholtz-berlin.de

Supplementary Information

Experimental details

Table 1. Counts of total, accidental and true coincidences for three Cu surfaces.

	Total Counts	Accidental Counts	True Counts
Cu(100)	686717	384233	302484
Cu(110)	1454830	899840	554990
Cu(111)	1208010	676705	531305

The counts in Tab. 1 are the total, accidental and true coincidence counts of the three Cu surfaces from the Auger photoelectron coincidence spectroscopy (APECS) measurements. The overall measurement time of Cu (100) surface was 8h 20min with an accidental to true ratio of $v_a/v_t = 1.27$. For the Cu(110) surface, the overall measurement the time was 12 h with an accidental to true ratio of $v_a/v_t = 1.62$. For Cu(111) surface measurement time was 15h with an accidental to true ratio of $v_a/v_t = 1.27$.

Energy calibration of Photoelectron and Auger Electron Spectra, and Calculation of Two-hole binding Energies

In Fig. 1, the energy calibration of the Cu(100) surface to its respective Fermi level is presented and described. The energy offset E_{PES}^{offset} used to convert measured kinetic energy in PES to calibrated kinetic energy is obtained by subtracting the measured kinetic energy of the Fermi edge (375.2 eV) from the photon energy (380 eV). This offset (4.8 eV for Cu 100) corresponds approximately to the spectrometer work function and additionally incorporates a small correction of the kinetic energy due to imperfect alignment of the sample with respect to the time-of-flight analyzer. Furthermore, the binding energy is set to zero at the Fermi edge of the metallic sample. The PES energy calibration for the other two surfaces Cu(110) and Cu(111) have been attained analogously. The E_{PES}^{offset} values obtained for Cu(100) is 4.8 $\pm$ 0.1 eV, Cu(110) is 4.65 $\pm$ 0.1 eV and for Cu(111) is 4.7 $\pm$ 0.1 eV. The Auger spectra of the three Cu surfaces Cu(100), Cu(110), and Cu(111) have been calibrated with an energy offset E_{AES}^{offset} of 4.2 $\pm$ 0.2 eV, which was obtained by adjusting the kinetic energy of the Cu M_3VV peak of the Cu(100) surface to the reported value of 61.0 eV from[1]. This energy offset value was found to be the same for all three surfaces. The step-by-step

conversion of kinetic energies to two-hole binding energies in the Photoelectron and Auger electron scale are described below also with the help of equations.

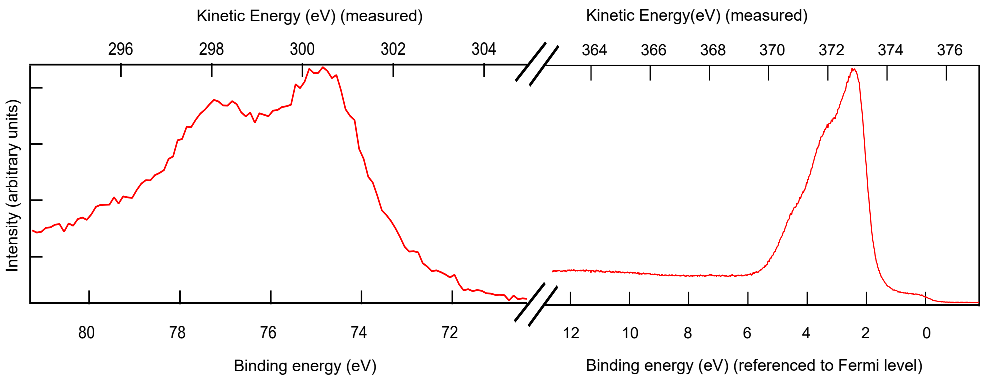

Figure 1. The energy calibration of Cu surface (100) to its respective Fermi level with the help of the valence band spectrum is shown. On the left side of the figure, the photoelectron spectrum is presented, with a kinetic energy scale on the top and binding energy at the bottom. The PES(kinetic energy) as measured spectrum is first calibrated to E_f= 0 eV, with the help of energy offset, obtained from the valence band and then converted to binding energy scale. The right side of the figure represents the valence band, with kinetic Energy as measured on the top and calibrated binding energy scale at the bottom. The valence band when converted to the binding energy scale, with the energy offset obtained shows Fermi to be at 0 eV, exhibiting the desired calibration for our measurements.

Following the calibration of AES and PES spectra, the two-hole binding energies were obtained for our energy sum spectra. The equation below gives the mathematical expression of the calculation of two-hole binding energies. E_{PES}^{offset} has been obtained from the valence band and E_{AES}^{offset} has been obtained from literature values, as described above. With the energy offset values obtained, the measured kinetic energy of PES and AES has been calibrated and converted further to two-hole binding energies.

$$E_{kin,PES}^{calib} = E_{kin,PES}^{measured} + E_{PES}^{offset}$$

$$E_{kin,AES}^{calib} = E_{kin,AES}^{measured} + E_{AES}^{offset}$$

$$E_{Bin}^{2H} = h\nu - E_{kin,PES}^{calib} - E_{kin,AES}^{calib}$$

Calculation of Depth-Dependent Intensity Contributions and Atomic Sheet Density

We calculated depth-dependent intensity contributions for our layer resolved fit model of the two-hole spectra. The facet specific depth profile of the signal arising from first and second layer of the Cu surface and the bulk is obtained by integrating the signal over the interval [p,q] using an exponential attenuation model. The intensity emitted from a layer of material between depths p and q is proportional to the integral as given by the Eq. 1:

$$I = I_0 \int_p^q e^{-x/\Delta} dx \tag{1}$$

Here, Δ is the mean escape depth (MED) of the material, denoted by $\Delta = \lambda \cdot cos\theta$, where the inelastic mean free path λ is obtained from[2,3] and is 0.497 nm for Auger electron kinetic energy (60 eV) and 0.785 nm for photoelectron kinetic energy (300 eV), applied under a Auger electron detection cone with a central angle of 49° and a photoelectron detection cone with a central angle of 59°, with respect to the sample surface normal.

The layer thickness (interlayer spacing) d of the three surface orientations (100,110 and 111) are calculated with $d_{hkl} = a/\sqrt{h^2+k^2+l^2}$ with the experimental bulk lattice constant $a = 0.359$ nm[4,5] for the three smallest allowed reciprocal lattice vectors of fcc copper with Miller indices h,k,l. For the interlayer spacings we obtain: $d_{200} = a/2 = 0.18$ nm for the 100 orientation, $d_{220} = a/(2\cdot\sqrt{2}) = 0.127$ nm for the 110 orientation and $d_{111} = a/\sqrt{3} = 0.207$ nm for the 111 orientation. These values are very similar to the inter layer spacings in our VASP calculations.

The integration limits (p,q) are based on the calculated layer thicknesses and tabulated for the first two layers of the Cu surfaces and bulk in Tab.2. Further, the atomic sheet densities (No of atoms/unit Area) of each layer were calculated from the

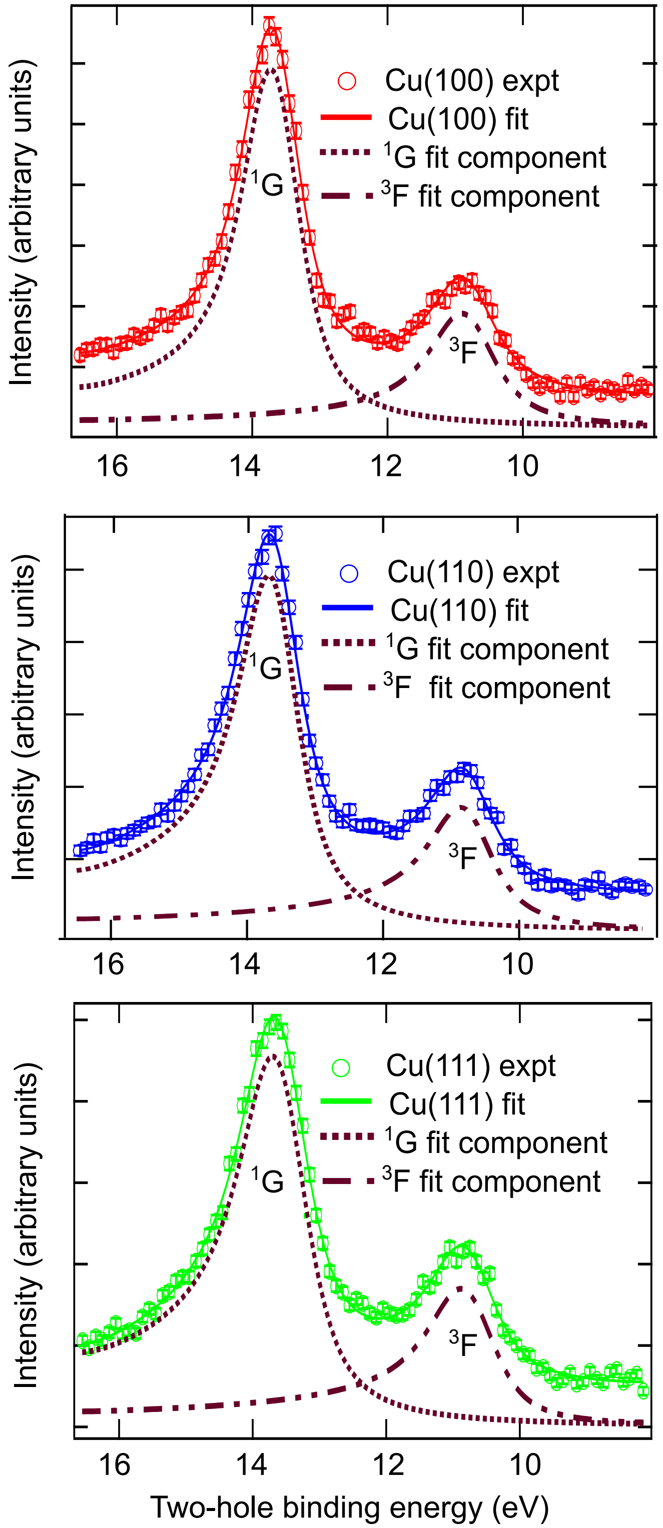

Figure 2. The ^{1}G and ^{3}F fit components (Doniach- Sunjic lineshapes), employed to make a completely unconstrained fit, to the experimental data of Cu(100), Cu(110) and Cu(111) surfaces, are presented here with the fits obtained.

lattice parameters derived from VASP and are found to be constant for all layers of each particular surface orientation. The depth dependent intensities (in %) from the first layer, second layer, and the bulk (including the third layer) were determined by multiplying the respective intensities from the exponential attenuation model with the atomic sheet densities. The intensity

values are tabulated in the Tab.3.

Table 2. Integration limits (p,q) in nm of Layer 1,2 and Layer 3 merged to bulk for three Cu surfaces obtained by depth profiling of each layer

	Cu(100)		Cu(110)		Cu(111)	
	p	q	p	q	p	q
Layer 1	0	0.180	0	0.127	0	0.207
Layer 2	0.180	0.360	0.127	0.254	0.207	0.414
Layer 3+ Bulk	0.360	∞	0.254	∞	0.414	∞

Table 3. Depth-dependent Intensity (in %) for Layer 1, 2 and Layer 3 merged to bulk for the Cu surfaces, obtained by calculating atomic sheet density and depth profiling from each layer

Facets	(Layer 1)	(Layer 2)	(Layer 3)+(bulk)
Cu(100)	63.2%	23.3%	13.5%
Cu(110)	50.6%	25%	24.4%
Cu(111)	68.3%	21.7%	10%

Table 4. Relative Intensity ratios of Layer 1, Layer 2 and Layer 3 + bulk for the three Cu surfaces obtained by atomic sheet density and depth profiling of each layer

	Relative Intensity (Layer 1)/(Layer 3+Bulk)	Relative Intensity (Layer 2)(Layer 3+Bulk)
Cu(100)	4.68	1.73
Cu(110)	2.07	1.02
Cu(111)	6.83	2.17

From the Tab.3, we determine intensity ratio of Layer 1 relative to Layer 3 + Bulk and intensity ratio of Layer 2 relative to Layer 3 + Bulk, tabulated in Tab.4. These depth-dependent relative ratios of intensity (used as input parameters) with layer-dependent energy shifts obtained from calculations and Doniach-Sunjic line shapes, build up the model for atomic contributions of different layers of Cu surfaces.

Determination of ^1G peak height for normalization with unconstrained fitting

To enhance the accuracy of normalization of Cu $3d^8 4s^2$ atomic multiplets of the Cu(100), Cu(110) and Cu(111) surfaces, we use a data smoothing method. A completely unconstrained fit of the ^1G and ^3F fit components (Doniach-Sunjic lineshapes) were applied to the experimental data of the Cu(100), (110) and (111) surfaces individually and intensity values of ^1G multiplets for all Cu surfaces were obtained. The peak intensity values obtained were further used to normalize the fit and data of the Cu surfaces. In Fig. 2 we present the ^1G and ^3F fit components (Doniach-Sunjic lineshapes), with the fits obtained for the Cu(100), Cu(110), and Cu(111) surfaces. All fitting procedures are done with (Igor Pro Software Version 8.0 (Windows)).

Surface signal from Layer 1 for Cu(111) surface in the 2p-L edge and 3p-M edge

As the electron intensity of the non-scattered electrons dI from a certain depth z measured vertically from the surface[6] is:

$$dI \propto \exp\left(\frac{-z}{\Delta}\right) \cdot dz \qquad (2)$$

Here, Δ is mean escape depth (MED) of the Cu(111) surface, obtained from $\Delta = \lambda \cdot cos\theta$, where the inelastic mean free path λ is obtained from[2,3] and is 0.497 nm for Auger electron kinetic energy (60 eV) and 0.785 nm for photoelectron kinetic energy (300 eV), applied under a Auger electron detection cone with a central angle of 49° and a photoelectron detection cone with a central angle of 59° for Cu 3p-MVV edge. For the Cu 2p-LVV edge the inelastic mean free path λ is also obtained from[2,3] and is 1.56 nm for Auger electron kinetic energy (912 eV) and 0.599 nm for photoelectron kinetic energy (163 eV), applied under a Auger electron detection cone with a central angle of 49° and a photoelectron detection cone with a central angle of 59°. And the depth dependent electron pair intensity dI_{pair} of non-scattered electron pairs is the product of the individual probabilities,

we have, the intensity of non-scattered electron pairs[6] given as Eq. 3 :

$$dI_{\text{pair}} \propto \exp\left(\frac{-z}{\lambda_{\text{PE}} \cdot \cos\theta_{\text{PE}}}\right) \cdot \exp\left(\frac{-z}{\lambda_{\text{AE}} \cdot \cos\theta_{\text{AE}}}\right) \cdot dz \tag{3}$$

the MED of the pairs of Auger and photoelectrons is then:

$$\Delta_{\text{pair}} = \frac{\Delta_{PE} \cdot \Delta_{AE}}{\Delta_{PE} + \Delta_{AE}} \tag{4}$$

And with this Eq. 4, for Cu 3p-MVV edge Auger and photoelectron pairs MED is 0.17 nm and for Cu 2p-LVV edge Auger and photoelectron pairs MED is 0.22 nm. If the surface layer is to have a rigid thickness d_s, then integrating Eq. 2 over the surface layer gives $I_S = (I_S + I_B) \cdot (1 - \exp(\frac{d_s}{\Delta}))$ and with the fractional surface intensity $f = \frac{I_S}{I_S + I_B}$ we have:

$$f = 1 - \exp\left(-\frac{d_s}{\Delta}\right) \tag{5}$$

Using this Eq. 5, with MED, Δ and surface layer thickness d_s= 0.21 nm for Cu(111) surface, we obtain the surface contribution from Cu 3p- MVV edge and Cu 2p-MVV edge. The surface signal contribution from Cu 3p-MVV edge is 71% and for Cu 2p-LVV edge is 61.5 %.

Arrangement of Ga impurities in outermost surface layers and inner layers in the Cu slab

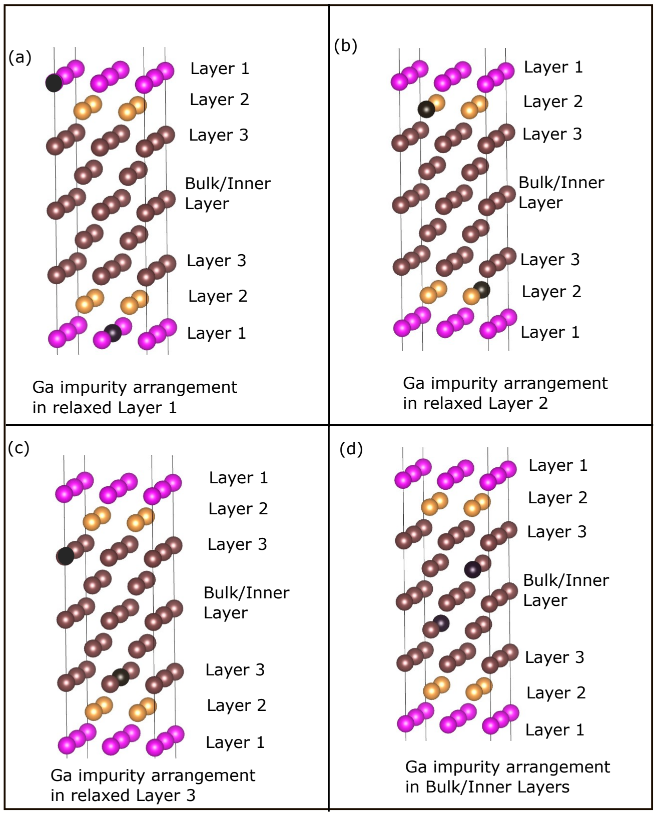

Figure 3. The arrangement of Ga impurities on both surfaces of the Cu(100) slab for the outermost layers: (a) Layer 1 in pink, (b) Layer 2 in orange, (c) Layer 3 in brown and the bulk/inner layers (d) also in brown.

The arrangement of Ga impurities in the 2*2 slab of the Cu(100) surface in the outermost layers 1, 2, 3 and bulk/inner layers are shown with pictorial representation in Fig. 3 (a), (b), (c) and (d), respectively. Based on the (Z+2) SCLS, Layer 3

converges to bulk like values and is therefore integrated into the bulk. Thus, Layer 3 is represented in brown colored atoms along with bulk/inner layers. For the outermost surface layers Ga impurities were placed symmetrically at both surfaces of each slab, see Fig. 3 (a) for Layer 1, Fig. 3 (b) for Layer 2 and Fig. 3 (c) for Layer 3. For the bulk/inner layers of the slab, the Ga impurities are placed at maximum possible distance from each other, see Fig. 3 (d). The Ga atoms are arranged in similar pattern in the slabs of Cu(110) and Cu(111). For the 4*4 slab model, the Ga impurities are arranged alike as in the 2*2 slabs for the three Cu surfaces.

References

1. Powell, C., Erickson, N. & Jach, T. Accurate determination of the energies of auger electrons and photoelectrons from nickel, copper, and gold. *J. Vac. Sci. Technol.* **20**, 625–625 (1982).

2. Powell, C. J. Practical guide for inelastic mean free paths, effective attenuation lengths, mean escape depths, and information depths in X-ray photoelectron spectroscopy. *J. Vac. Sci. & Technol. A* **38**, 023209 (2020).

3. Shinotsuka, H., Tanuma, S., Powell, C. J. & Penn, D. R. Calculations of electron inelastic mean free paths. X. data for 41 elemental solids over the 50 eV to 200 kev range with the relativistic full Penn algorithm. *Surf. Interface Analysis* **47**, 871–888 (2015).

4. Kittel, C. & McEuen, P. *Introduction to Solid State Physics* (John Wiley & Sons, 2018).

5. Hermann, K. *Crystallography and Surface Structure: An Introduction for Surface Scientists and Nanoscientists* (John Wiley & Sons, 2016).

6. Kühn, D. *et al.* Enhanced Surface Determination beyond Photoemission via Auger Photoelectron Coincidence Spectroscopy. *The J. Phys. Chem. Lett.* **15**, 8161–8166 (2024).
